# Nemolizumab for the treatment of refractory primary localized cutaneous amyloidosis in a nonatopic patient

**DOI:** 10.1016/j.jdcr.2025.04.006

**Published:** 2025-04-22

**Authors:** Gabriela Soto-Canetti, Jiwon Park, Avi Bitterman, Jordan Talia

**Affiliations:** a Department of Dermatology, Icahn School of Medicine at Mount Sinai, New York, New York; b Ponce Health Sciences University School of Medicine, Ponce, Puerto Rico; c University of Texas Health Science Center at San Antonio, Long School of Medicine, San Antonio, Texas

**Keywords:** atopic dermatitis, lichen amyloidosis, macular amyloidosis, nemolizumab, primary localized cutaneous amyloidosis

## Introduction

Primary localized cutaneous amyloidosis (PLCA) is a chronic disorder characterized by the extracellular deposition of amyloid proteins in the skin, with lichen amyloidosis (LA) and macular amyloidosis as the most common subtypes. Clinically, LA presents as intensely pruritic, hyperkeratotic, flesh-colored to hyperpigmented plaques and papules, predominantly affecting the extensor surfaces, whereas macular amyloidosis presents as pruritic, hyperpigmented patches. The combined presentation termed biphasic amyloidosis.^1^ A chronic itch-scratch cycle is thought to contribute to amyloid deposition of keratin intermediate filament proteins.^1^ The first-line treatment for LA involves topical or intralesional corticosteroids and oral antihistamines, with laser therapy, phototherapy, and cyclosporine reserved for refractory cases.^1^ Herein, we report a case of PLCA and no atopic history who was successfully treated with nemolizumab, a monoclonal antibody against the interleukin (IL)-31 receptor alpha (IL-31Rα).

## Case presentation

**Fig 1:**
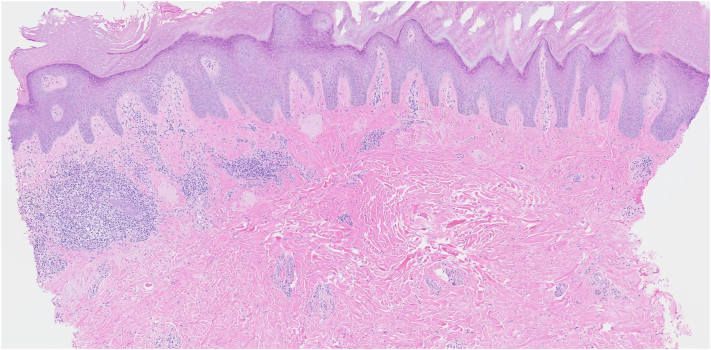
Beneath an acanthotic epidermis, there are globules of an amphophilic material within a background of lymphogranulomatous inflammation. (Hematoxylin-eosin stain; original magnification: ×5.)

**Fig 2:**
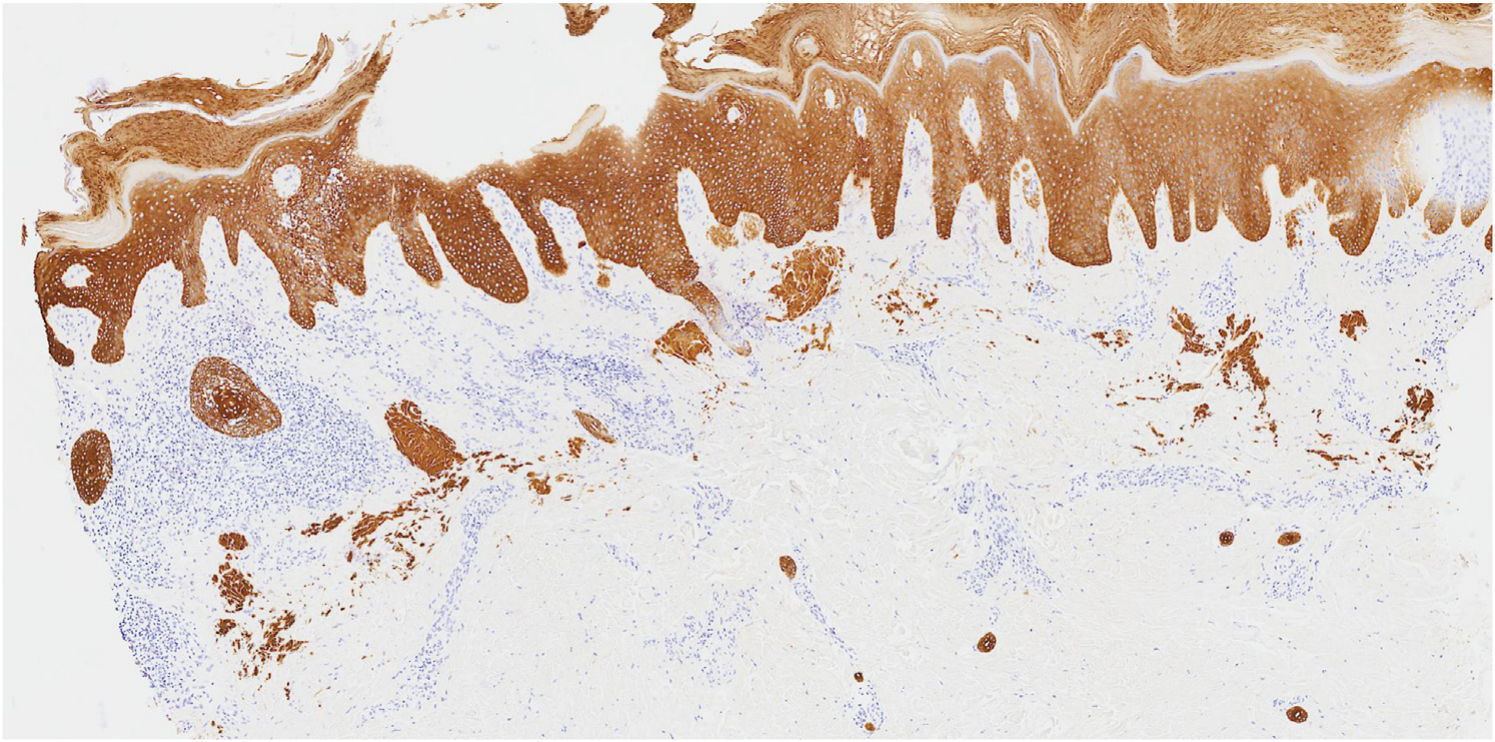
Keratin 903 stain highlights the amphophilic globular material, indicating keratin derived amyloid consistent with lichen amyloidosis. (Keratin 903 stain; original magnification: ×5.)

**Fig 3:**
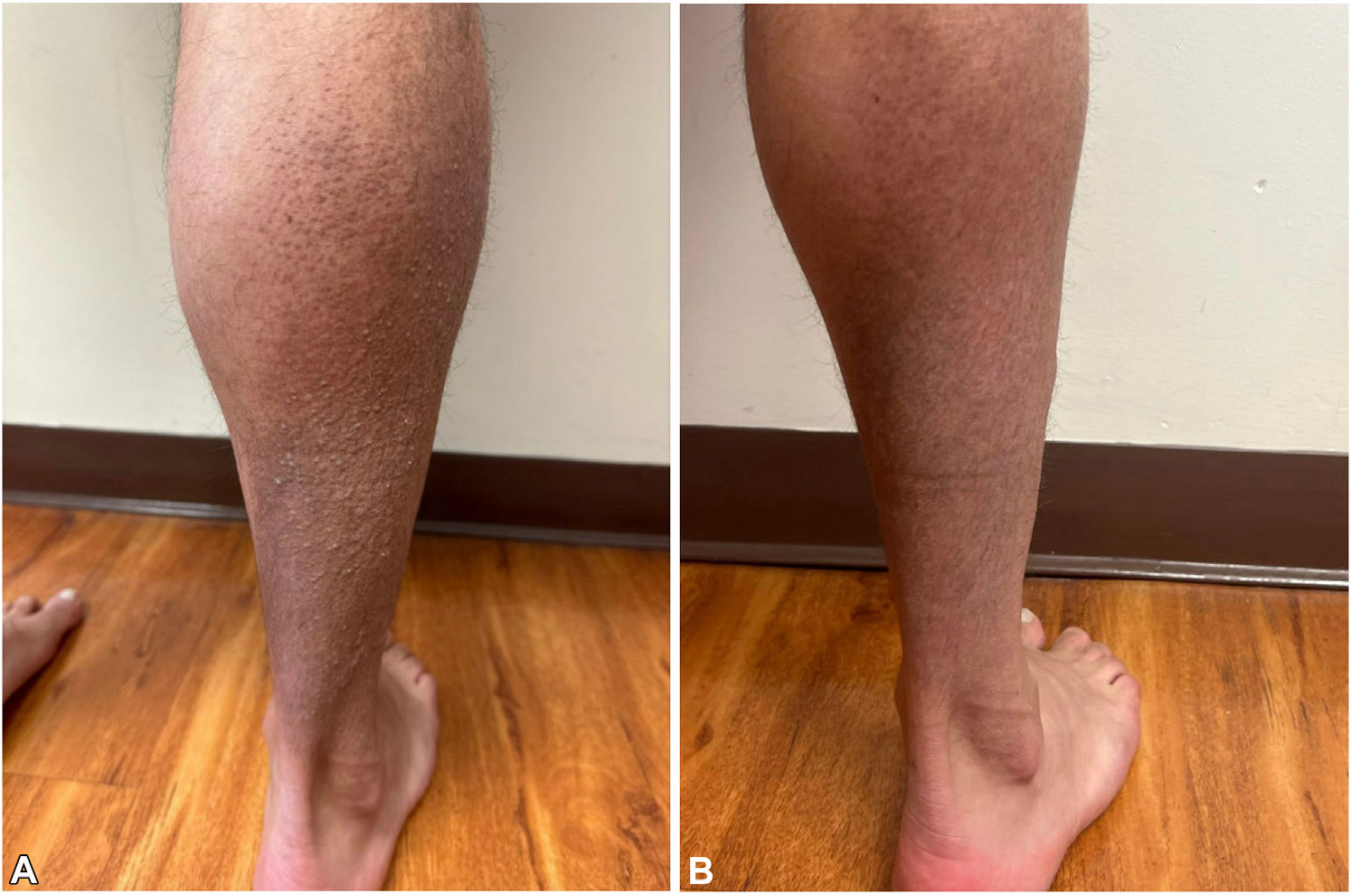
**A,** Physical examination on initial presentation demonstrating multiple hyperkeratotic, brown plaques and papules on the right posterior lower extremity. **B,** Results after 2 months of therapy with nemolizumab demonstrating flattening of papules and plaques and decreased hyperpigmentation.

A 44-year-old man presented with a 30-year history of a progressively worsening pruritic eruption localized to the extremities. Physical examination revealed multiple hyperkeratotic, brown to flesh-colored papules and plaques on the right side of the lower extremity with hyperpigmented patches on the right side of the upper extremity and buttocks. Histopathologic evaluation from biopsies performed in 2006 and 2017 had confirmed a diagnosis of LA. The patient denied a history of atopy, and the serum immunoglobulin E was 5 IU/mL (reference range, 6-495 IU/mL). The patient reported intermittent use of clobetasol cream and triamcinolone ointment for several years, alongside trials of narrowband UVB phototherapy, which provided partial relief of pruritus. A repeat punch biopsy of the right side of the lower extremity demonstrated background lymphogranulomatous inflammation with keratin 903 staining reaffirming the diagnosis (Figs 1 and 2). Background lymphogranulomatous inflammation was favored to be secondary to chronic scratching, with staining confirming amyloid deposition. Dupilumab was initiated at standard prurigo nodularis dosing. After 5 months of therapy, the patient had persistent pruritus with only mild improvement in the hyperkeratosis of cutaneous lesions and subsequently developed arthralgia of the wrists. Dupilumab was discontinued and nemolizumab was initiated with a loading dose of 60 mg subcutaneously and 60 mg every 4 weeks thereafter based on the patient’s weight. Five days after initiation of nemolizumab, the patient reported immediate relief of pruritus. Two months into nemolizumab therapy, there was sustained resolution of pruritus, with markedly improved hyperkeratosis of cutaneous lesions on the LA compared with baseline (Fig 3, *A, B*). The patient’s arthralgia began to subside, secondary to dupilumab discontinuation.

## Discussion

The exact pathogenesis of LA and macular amyloidosis remains unclear; however, amyloid deposition in the papillary dermis is thought to derive from degenerating basal keratinocytes in the epidermis, which are phagocytosed to produce amyloid K.^1^

IL-31, a T helper 2-derived cytokine known for its role in pruritus, has been implicated in the pathogenesis of PLCA.^2^ Studies of patients with familial PLCA have identified mutations in the oncostatin M receptor (OSMR) gene that encodes the OSMRβ subunit and the IL-31Rα subunit gene.^3^ Both mutations are believed to trigger signaling abnormalities resulting in keratinocyte apoptosis and pruritus.^3^ A study by Tey et al^2^ found increased epidermal expression of OSMRβ and IL-31Rα in PLCA cases. Furthermore, 1 case report also demonstrated upregulation of IL-31 gene expression in lesional skin of a patient with sporadic LA.^3^

Nemolizumab, an IL-31Rα human monoclonal antibody, is approved for atopic dermatitis (AD) and prurigo nodularis. Efficacy of both nemolizumab and dupilumab has been shown in patients with PLCA and AD, with a recent report showing dupilumab success in a non-AD patient.^4^, ^5^, ^6^ To our knowledge, this is the first report of PLCA in a nonatopic patient successfully treated with nemolizumab and with rapid and lasting resolution of pruritus. Further large-scale studies are required to demonstrate efficacy and safety in patients with PLCA, both with and without AD.

## Conflicts of interest

Dr Talia has served as a consultant for Abbvie, Arcutis Biotherapeutics, Bristol-Meyers Squibb, Calliditas Therapeutics, Johnson & Johnson, LEO Pharma, Primus Pharmaceuticals, Sanofi Genzyme, Stifel Financial, and UCB and serves as an investigator for LEO Pharma and Sanofi. Authors Soto-Canetti, Park, and Bitterman have no conflicts of interest to declare.
